# Trauma-sensitive obstetrics: Potential for optimization from the providers’ perspectives

**DOI:** 10.18332/ejm/219322

**Published:** 2026-06-23

**Authors:** Louisa Horvath, Silke Pawils

**Affiliations:** 1 University Medical Center Hamburg-Eppendorf (UKE), Hamburg, Germany

**Keywords:** midwifery care, trauma-sensitive obstetrics, provider perspective, obstetric care quality, inpatient maternity care

## Abstract

**INTRODUCTION:**

Traumatic birth experiences are increasingly recognized as a significant issue in maternity care. While many studies focus on the perspectives of birthing persons, little is known about how healthcare providers perceive the causes of traumatic births and possible strategies to prevent them. This study explores obstetric care providers’ perceptions of factors contributing to traumatic birth experiences and their suggestions for improving trauma sensitive obstetric care.

**METHODS:**

A convergent parallel mixed methods design was used. An online survey among obstetric care providers in Germany was conducted between October 2024 and February 2025 (n=102). Quantitative data were analyzed descriptively and using logistic regression models. In addition, four expert interviews were conducted and analyzed using qualitative content analysis. Findings from both strands were integrated during interpretation.

**RESULTS:**

Most respondents defined traumatic birth experiences primarily through loss of control (85.3%) and negative emotions (75.5%). Lack of communication and information was identified as the most important contributing factor (97.1%), followed by lack of emotional support (89.2%). Emotional support was rated as the most important preventive measure (mean=4.86; SD=0.51). The main barriers to trauma sensitive care were lack of time (mean=4.52; SD=0.81) and staff shortages (mean=4.35; SD=0.83). Interview findings highlighted structural challenges, the importance of reflective practice among caregivers, and the need for systematic training in trauma sensitive communication.

**CONCLUSIONS:**

The findings highlight the need to strengthen trauma sensitive approaches in obstetric care through improved communication, emotional support, and shared decision making. In addition to structural improvements such as adequate staffing, regular training and reflective practice among providers may help reduce traumatic birth experiences.

## INTRODUCTION

Childbirth, while often positive, can be experienced as traumatic^1^. Studies report 9–50% of births as traumatic, with about 5% leading to postpartum post-traumatic stress disorder (PP-PTSD)^2^. In high-risk groups, prevalence rises to 15.7%^3^. Symptoms such as flashbacks, nightmares, and severe stress may persist for years, negatively affecting quality of life and parent-child bonding^1,2,4,5^.

Trauma may result from events during birth (primary traumatization) or from re-traumatization based on previous experiences, often linked to past violence^1,2,6-8^. Studies show that recognizing at-risk individuals reduces re-traumatization and improves care outcomes^5,9^. It is important to recognize that the birthing person’s subjective experience may differ significantly from the perception of the attending healthcare providers^2,10,11^.

In recent years, research has increasingly examined the perspectives of birthing persons on traumatic birth experiences. Central triggers reported a lack of communication, insufficient informed consent, and inadequate emotional support^9,10,12-15^.

The World Health Organization (WHO) has therefore defined the concept of ‘Respectful Maternity Care’ (RMC)^16^. National associations, such as the German Association of Midwives and the German Society of Gynecology and Obstetrics (DGGG), also call for trauma-sensitive practice and training^17,18^.

Despite the recommendations, concrete strategies for implementing trauma-sensitive care in Germany remain limited^16,19^. The German Society of Midwifery Sciences has emphasized the need for more research not only into prevalence and prevention, but also into effective approaches for caring for vulnerable pregnant persons, as well as the use of both qualitative and quantitative research designs^19^. To translate the WHO recommendations into practice, consistent implementation in healthcare policy and clinical structures is required^20^.

To date, most research has focused on the perspectives of birthing persons, with little attention to those of healthcare providers. Yet their role is crucial, as providers directly shape maternity care and may contribute to either supportive or traumatic experiences^21^. Further research is needed to address this gap and improve care structures.

This study investigates opportunities for optimizing inpatient obstetric care from a trauma-sensitive perspective. It focuses on the perceptions of midwives and physicians regarding factors contributing to traumatic birth experiences and their suggestions for preventing them.

## METHODS

### Study design

A convergent parallel mixed-methods design was used to capture both general patterns in providers’ perceptions and in-depth insights from experts in trauma-sensitive care^22^. Quantitative data collection was complemented by four expert interviews to provide a more comprehensive understanding. Integrating both strands enabled triangulation and was essential to capture the complexity of traumatic birth experiences.

### Quantitative data collection: online survey

An online survey was conducted in Germany from 28 October 2024 to 1 February 2025 in birth clinics, in which inpatient obstetric staff participated in an online questionnaire. The target group comprised gynecologists and midwives working in inpatient obstetric care. The questionnaire was administered via SoSciSurvey and developed by the research group based on literature on trauma-sensitive care and previous conceptual work^1-7,10-17,19,21^. In particular, item list of Salmon and Drew^23^ and the Child Birth Experience Questionnaire 2^24,25^ were used as a reference.

The sample was recruited by issuing QR codes on study information which was distributed in maternity wards in clinics throughout Germany. Study information was distributed to 210 clinics; however, participation occurred at the individual level, and the exact response rate at the clinic level could not be determined. The distribution of the clinics contacted was calculated in proportion to the population of the federal states, with attention to a balanced selection of clinics across different care levels (maximum care, specialized care, and basic and standard care).

A minimum target sample size of 35 participants per professional group (medicine/midwifery) was defined to enable meaningful between-group comparisons (α=0.05, power=0.80) and adequate power for association analyses. Participants were included until the required sample size was reached. Participants were included if they: 1) provided complete information on their occupational group, and 2) had a missing rate of <30%.

### Measures

Prior to publication, a pretest was conducted with two obstetric care providers (a physician and a midwife) to ensure clarity and feasibility. Since no standardized or validated instrument exists for the specific research question, items were self-developed to capture providers’ perceptions. The instruments included the following four main constructs.

*Understanding of traumatic birth experiences*

Definition

Participants were asked what they considered a ‘traumatic birth’. Predefined options (medical complications, loss of control/helplessness, negative emotions, other) were provided, allowing multiple selections. Additional aspects could be specified in a free-text field.

Estimated prevalence

Participants estimated the proportion of traumatic births in Germany by selecting predefined categories (very rare: 0–10%, occasional: 11–25%, frequent: 26–50%, very common: 51–75%).

Perceived consequences

Participants rated seven statements on long-term psychological and relational consequences of traumatic birth on a five-point Likert scale (1=do not agree at all, to 5=fully agree).

*The contributing and protective factors*

Causes

Participants indicated which factors they considered contributors to traumatic birth experiences (e.g. lack of communication, insufficient informed consent, insufficient emotional support, obstetric emergencies). Multiple responses were possible, plus a free-text option.

Importance of preventive measures

Participants rated the importance of seven measures (e.g. early identification of at-risk individuals, emotional support, mandatory training) on a five-point Likert scale (1=not at all important, to 5=very important).

*Current practice and barriers*

Implementation of trauma-sensitive care

The same seven measures were rated again, this time regarding their current implementation in participants’ institution (1=very poor, to 5=very good).

Barriers

Participants assessed the extent to which specific aspects (staff shortages, lack of time, financial resources, structural barriers) hindered trauma-sensitive care (Likert scale: 1=not at all, to 5=very strongly). Additional barriers could be described in free-text fields.

*The improvement strategies*

Solutions

Participants suggested possible solutions to the above obstacles in free-text fields.

Need for trauma-sensitive care

Finally, participants were asked whether they considered trauma-sensitive obstetric care necessary (yes, no, uncertain) and were able to provide reasoning in a free-text field.

Sociodemographic data were collected. This study was conducted in accordance with the CHERRIES checklist^26^ given in the Supplementary file.

### Qualitative data collection: expert interviews

To supplement and deepen the quantitative results, four guided interviews were conducted with experts from obstetrics, again represented by the two professional groups of physicians and midwives. Experts were defined as clinicians with extensive professional experience in obstetrics and documented engagement with trauma-sensitive care (e.g. training activities, publications, or clinical leadership roles). The interview guidelines were based on the questions from the online survey. Participants were purposively selected for their expertise in trauma-sensitive obstetrics or their critical perspectives on this topic. The interviews were conducted online, audio-recorded with consent, and transcribed verbatim. The interviews were conducted to complement the quantitative findings and provide illustrative expert perspectives rather than achieve theoretical saturation.

### Statistical analysis

Descriptive statistics were used to characterize the sample and reflect the content of the responses. A regression analysis was conducted to examine differences between occupational groups, using logistic regression models (binary and dichotomized). The covariate professional experience (in years) was included in the models. Since the study focused on adjusted associations, crude estimates are not reported here but were considered during model development. For the professional group, category 2 ‘midwives’ was chosen as the reference group, while the third category ‘>20 years’ was chosen for professional experience, so that all other categories are interpreted relative to this one. Statistical significance was set at p<0.05, and effect estimates are presented with 95% confidence intervals (95% CI). The statistical analysis was conducted using IBM SPSS Statistics 29.0.2.0. The interview data and the free text responses from the survey were transcribed using the MAXQDA Analytics Pro 24 program and analyzed using qualitative content analysis according to Kuckartz and Rädiker^27^, clustered in terms of content, and presented in the order of the most frequent mentions. Integration of quantitative and qualitative findings followed the logic of a convergent parallel design as described by Creswell and Plano Clark^22^. Results from both strands were compared, contrasted, and synthesized to identify convergences and complementary insights.

## RESULTS

### Sample description

Of 147 participants, 102 met the inclusion criteria: 66 midwives (64.7%) and 36 physicians (35.3%). The majority were female (98.0%), and 52.9% had 0–5 years of professional experience. The mean age was 35.72 years (SD=11.55; range: 22–65). Detailed sociodemographic characteristics are shown in Table 1.

**Table 1:** Sociodemographic characteristics of obstetric care providers in Germany by occupational group, a cross-sectional online survey on trauma-sensitive care, 2024–2025 (N=102)

*Characteristics*	*Physician (N=36)* *n (%)*	*Midwife (N=66)* *n (%)*	*Total (N=102)* *n (%)*
**Gender**			
Female	34 (94.4)	66 (100)	100 (98.0)
Male	2 (5.6)	n.a	2 (2.0)
Non-binary	n.a	n.a	n.a
Missing	0	0	0
**Federal state**			
Baden-Württemberg	2 (5.6)	5 (7.6)	7 (6.9)
Bavaria	3 (8.3)	6 (9.1)	9 (8.8)
Berlin	10 (27.8)	9 (13.6)	19 (18.6)
Brandenburg	2 (5.6)	2 (3.0)	4 (3.9)
Bremen	1 (2.8)	1 (1.5)	2 (2.0)
Hamburg	10 (27.8)	14 (21.2)	24 (23.5)
Hesse	n.a	4 (6.1)	4 (3.9)
Mecklenburg-Western Pomerania	2 (5.6)	n.a	2 (2.0)
Lower Saxony	2 (5.6)	10 (15.2)	12 (11.8)
North Rhine-Westphalia	2 (5.6)	8 (12.1)	10 (9.8)
Rhineland-Palatinate	2 (5.6)	n.a	2 (2.0)
Saarland	n.a	2 (3.0)	2 (2.0)
Saxony	n.a	2 (3.0)	2 (2.0)
Schleswig-Holstein	n.a	2 (3.0)	2 (2.0)
Missing	0	1 (1.5)	1 (1.0)
**Place of work**			
Clinic with maximum care	23 (63.9)	36 (54.5)	59 (57.8)
Clinic for specialized care	8 (22.2)	10 (15.2)	18 (17.6)
Clinic for basic and standard care	n.a	3 (4.5)	3 (2.9)
Birth center	1 (2.8)	4 (6.1)	5 (4.9)
Other*	4 (11.1)	13 (19.7)	17 (16.7)
Missing	0	0	0
**Catchment area**			
Urban	23 (63.9)	32 (48.5)	55 (53.9)
Rural	2 (5.6)	4 (6.1)	6 (5.9)
Both	11 (30.6)	30 (45.5)	41 (40.2)
Missing	0	0	0
**Professional experience** (years)			
0–5	20 (55.6)	34 (51.5)	54 (52.9)
6–20	8 (22.2)	19 (28.8)	27 (26.5)
>20	8 (22.2)	13 (19.7)	21 (20.6)
Missing	0	0	0
**Age** (years)			
Mean	37.97	34.51	35.72
SD	11.482	11.495	11.550
Range	26–65	22–63	22–65
Missing	2 (5.6)	3 (4.5)	5 (4.9)

n.a: not available.

* Additionally freelance, home birth midwife, psychotherapeutic practice.

### Understanding of traumatic birth experiences

*Definition*

Most participants (85.3%; n=87) defined a traumatic birth as characterized by ‘loss of control/helplessness’. A quarter (24.5%; n=25) gave additional answers in the free-text field, most commonly ‘the subjective experience of the birthing person’.

*Estimated prevalence*

Of the physicians, 63.9% (n=23) estimated traumatic births as being ‘occasional’ (11–25% of all births), whereas 51.5% (n=34) of midwives considered them ‘frequent’ (26–50% of all births).

*Perceived consequences*

The highest agreement was with the statement that psychological consequences are underestimated in care practice (mean=4.27; SD=0.997; range: 1–5). The discrepancy between the subjective experience of a traumatic birth by the person giving birth and the perception of the care providers was rated as relevant by both professional groups (mean=4.19; SD=0.80; range: 1–5). The majority rejected the statement that births never lead to PTSD (mean=1.18; SD=0.49; range: 1–4).

### Contributing and protective factors

*Causes*

‘Lack of communication/education’ was identified as the main contributing factor by nearly all respondents [100% (n=36) of physicians, 95.5% (n=63) of midwives]. In free-text responses (18.6%; n=19), additional systemic causes were named, including staff shortages, time constraints, and language barriers.

*Importance of preventive measures*

Preventive measures were consistently rated as highly important, with emotional support receiving the highest rating (mean=4.86; SD=0.510; range: 1–5).

### Current practice and barriers

*Implementation of trauma-sensitive care*

The implementation of emotional support was rated highest overall (mean=3.80; SD=0.86; range: 1–5). By contrast, mandatory training on trauma-sensitive obstetrics was rated low (mean=1.79; SD=1.10; range: 1–5).

*Barriers*

The most strongly perceived barrier was ‘lack of time’ (mean=4.52; SD=0.805; range: 1–5). Free-text responses clustered into two groups: 1) systemic problems (e.g. lack of trauma-sensitive training, absence of supervision, non-adherence to guidelines); and 2) interpersonal challenges (e.g. unsympathetic staff, dealing with strangers in extreme situations, lack of trauma-sensitive understanding).

### Improvement strategies

*Solutions*

The most frequently mentioned solution to the staff shortage was the ‘1:1 care ratio’, followed by ‘better pay’ and ‘more attractive working conditions’.

For time constraints, ‘more staff’ was the most frequent solution. For financial resources, participants suggested reforms in the remuneration system for obstetrics. In terms of solutions to structural hurdles, two response categories were identified, which accounted for a large proportion of the proposed solutions. Firstly, ‘better organization and infrastructure’ was mentioned. Secondly, ‘education and awareness-raising’ was mentioned as an important aspect.

*Need for trauma-sensitive obstetric care*

Most respondents endorsed the necessity of trauma-sensitive care (87.3%; n=86). Reported reasons included long-term psychological consequences for birthing persons, children, and couples, as well as the high number of traumatized individuals.

Table 2 provides a detailed summary of the descriptive survey findings reported in the sections above.

**Table 2:** Perceptions of traumatic birth experiences and trauma-sensitive care among obstetric care providers in Germany, a cross-sectional online survey, 2024–2025 (N=102)

*Perceptions*	*Physician (N=36)* *n (%)*	*Midwife (N=66)* *n (%)*	*Total (N=102)* *n (%)*
**Definition of traumatic births**			
Medical complication	18 (50.0)	28 (42.4)	46 (45.4)
Loss of control/helplessness	33 (91.7)	54 (81.8)	87 (85.3)
Negative emotions	30 (83.3)	47 (71.2)	77 (75.5)
Others	5 (13.9)	20 (30.3)	25 (24.5)
Missing	0	0	0
**Proportion of traumatic births**			
Very rare (0–10%)	1 (2.8)	7 (10.6)	8 (7.8)
Occasional (11–25%)	23 (63.9)	21 (31.8)	44 (43.1)
Frequent (26–50%)	10 (27.8)	34 (51.5)	44 (43.1)
Very common (51–75%)	2 (5.6)	4 (6.1)	6 (5.9)
Missing	0	0	0
**Cause of traumatic birth**			
Birth emergencies and complications	28 (77.8)	44 (66.7)	72 (70.6)
Lack of emotional support	31 (86.1)	60 (90.9)	91 (89.2)
Pain	23 (63.9)	27 (40.9)	50 (49.0)
Surgical intervention	21 (58.3)	42 (63.6)	63 (61.8)
Lack of communication/information	36 (100)	63 (95.5)	99 (97.1)
Previous traumatic experience	30 (83.3)	50 (75.8)	80 (78.4)
Lack of privacy	22 (61.1)	35 (53.0)	57 (56.0)
Other	6 (16.7)	13 (19.7)	19 (18.6)
Missing	0	0	0
	***Mean (SD) range***	***Mean (SD) range***	***Mean (SD) range***
**Consequences of traumatic births^a^**			
No long-term psychological consequences	1.94 (1.12) 1–5	1.94 (1.06) 1–5	1.94 (1.075) 1–5
No PTSD after birth	1.11 (0.32) 1–2	1.21 (0.57) 1–4	1.18 (0.496) 1–4
No re-traumatization	1.64 (0.93) 1–4	1.61 (0.93) 1–5	1.62 (0.925) 1–5
No influence on parent–child relationship	1.67 (0.98) 1–4	1.83 (1.02) 1–5	1.77 (1.004) 1–5
No influence on couple relationship	1.61 (0.80) 1–4	1.74 (0.83) 1–5	1.70 (0.818) 1–5
Psychological consequences are underestimated	4.33 (0.83) 2–5	4.24 (1.08) 1–5	4.27 (0.997) 1–5
Discrepancy in perception	4.39 (0.60) 3–5	4.08 (0.88) 1–5	4.19 (0.805) 1–5
Missing n (%)	0	5 (7.5)	5 (4.9)
**Implementation of trauma-sensitive measures^b^**			
Early identification of people at risk	3.22 (1.25) 1–5	3.00 (1.28) 1–5	3.08 (1.262) 1–5
Improving communication and education	3.23 (1.09) 1–5	3.26 (1.09) 1–5	3.25 (1.086) 1–5
Strengthening self-determined decision-making	3.33 (0.99) 1–5	3.37 (1.02) 1–5	3.36 (1.006) 1–5
Emotional support from caregivers	3.92 (0.81) 1–5	3.74 (0.89) 1–5	3.80 (0.860) 1–5
Consistently obtaining consent for measures	3.72 (1.11) 1–5	3.77 (1.08) 1–5	3.75 (1.086) 1–5
Offer of psychological support	3.28 (1.21) 1–5	3.00 (1.33) 1–5	3.10 (1.291) 1–5
Training on trauma-sensitive obstetrics	1.86 (1.00) 1–5	1.75 (1.16) 1–5	1.79 (1.100) 1–5
Missing n (%)	2 (5.6)	14 (21.2)	16 (15.6)
**Importance of trauma-sensitive measures^c^**			
Early identification of people at risk	4.61 (0.69) 2–5	4.69 (0.71) 1–5	4.66 (0.698) 1–5
Improving communication and education	4.69 (0.86) 1–5	4.77 (0.63) 1–5	4.75 (0.713) 1–5
Strengthening self-determined decision-making	4.42 (0.87) 2–5	4.53 (0.85) 1–5	4.49 (0.853) 1–5
Emotional support from caregivers	4.86 (0.42) 3–5	4.86 (0.56) 1–5	4.86 (0.510) 1–5
Consistently obtaining consent for measures	4.69 (0.58) 3–5	4.80 (0.59) 1–5	4.75 (0.586) 1–5
Offer of psychological support	4.53 (0.65) 3–5	4.58 (0.66) 1–5	4.56 (0.654) 1–5
Training on trauma-sensitive obstetrics	4.36 (1.05) 1–5	4.42 (0.88) 1–5	4.40 (0.936) 1–5
Missing n (%)	0	3 (4.5)	3 (2.9)
**Obstacle to trauma-sensitive measures^d^**			
Staff shortage	4.22 (0.87) 2–5	4.42 (0.81) 1–5	4.35 (0.828) 1–5
Lack of time	4.36 (0.87) 1–5	4.61 (0.76) 1–5	4.52 (0.805) 1–5
Financial resources	3.52 (1.23) 1–5	3.44 (1.10) 1–5	3.47 (1.144) 1–5
Structural hurdles	3.86 (1.02) 1–5	3.92 (1.00) 1–5	3.90 (1.000) 1–5
Further obstacles	4.19 (0.75) 2–5	4.14 (0.85) 2–5	4.16 (0.800) 2–5
Missing n (%)	13 (36.1)	48 (72.7)	61 (59.8)
	***n (%)***	***n (%)***	***n (%)***
**Need for trauma-sensitive obstetric care**			
Yes, because	33 (91.7)	56 (84.8)	89 (87.3)
No, because	n.a	1 (1.5)	1 (0.9)
Uncertain, because	1 (2.8)	5 (7.6)	6 (5.8)
Missing	2 (5.6)	4 (6.1)	6 (5.9)

a 1=do not agree at all, to 5=fully agree.

b 1=very poor, to 5=very good.

c 1=not at all important, to 5=very important.

d 1=not at all, to 5=very strongly. n.a: not available.

### Factors influencing the definition and perceived need for trauma-sensitive care

Logistic regression analyses revealed no statistically significant effects of occupational group or professional experience on either the definition of traumatic birth experiences or the perceived need for trauma-sensitive obstetric care. Some non-significant trends were observed: respondents with >20 years of experience more often emphasized ‘medical complications’ (AOR=0.51; 95% CI: 0.18–1.43; p=0.20) as defining features, whereas ‘loss of control’ and ‘negative emotions’ were more likely to be chosen by participants with little to medium professional experience. Less experienced professionals showed a tendency towards stronger agreement with the need for trauma-sensitive care (AOR=4.07; 95% CI: 0.82–20.20; p=0.08). However, the wide confidence interval indicates that the estimate is subject to a high degree of uncertainty. A complete overview of the regression analyses is provided in Table 3.

**Table 3:** Logistic regression analyses of factors associated with obstetric care providers’ definitions of traumatic birth and perceived need for trauma-sensitive obstetric care, a cross-sectional online survey in Germany, 2024–2025 (N=102)

*Dependent variable*	*Predictor*	*p*	*AOR*	*95% CI*
**Medical complication**	Occupational group (ref. midwives)	0.468	1.357	0.595–3.094
	Professional experience (years) (ref. >20)			
	0–5	0.204	0.515	0.185–1.434
	6–10	0.403	0.614	0.194–1.948
**Loss of control/helplessness**	Occupational group (ref. midwives)	0.161	2.623	0.681–10.106
	Professional experience (years) (ref. >20)			
	0–5	0.794	1.192	0.318–4.467
	6–10	0.210	3.215	0.519–19.925
**Negative emotions**	Occupational group (ref. midwives)	0.156	2.119	0.751–5.978
	Professional experience (years) (ref. >20)			
	0–5	0.405	1.607	0.527–4.904
	6–10	0.209	2.374	0.617–9.137
**Other**	Occupational group (ref. midwives)	0.066	0.359	0.121–1.069
	Professional experience (years) (ref. >20)			
	0–5	0.306	0.552	0.177–1.722
	6–10	0.323	0.516	0.139–1919
**Need for traumasensitive obstetric care**	Occupational group (ref. midwives)	0.367	1.898	0.471–7.641
	Professional experience (years) (ref. >20)			
	0–5	0.086	4.070	0.820–20.203
	6–10	0.842	0.864	0.207–3611

AOR: adjusted odds ratio.

### Supplementary qualitative expert assessments

*Providers’ understanding of traumatic birth experiences*

The experts described traumatic birth experiences in heterogeneous ways. Two referred to established definitions of trauma, such as the diagnostic criteria of PTSD or the definition of trauma by Fischer and Riedesser^28^. At the same time, all emphasized the importance of the subjective experience of the birthing person and the risk of re-traumatization. The experts were critical of the correlation between obstetric complications and the severity of the traumatic experience, as a physiological birth can certainly be traumatizing. One expert also differentiated between traumatization and experiences that were disappointing but did not meet clinical criteria for trauma:

*‘… can it sometimes just be sadness that something didn’t go the way you wanted it to?’* (Expert 3)

*Experience of trauma in obstetrics*

The experts had experienced traumatic births in their work as midwives or physicians in obstetrics. However, it became clear in the interviews that these experiences were only seen and perceived as such in retrospect. It was clearly stated that the recognition and perception of trauma in obstetrics is still new:

*‘I have 24 years of obstetrics behind me, and it has to be said that the topic that a birth can be traumatic was an absolute no-go.’* (Expert 2)

*Factors contributing to traumatic birth experiences*

The experts identified multiple factors contributing to traumatic births. One expert referred to ‘3-C factors’ (control, participation, continuity) of Hundley et al.^29^, noting that the absence of these elements can lead to traumatization. Structural and organizational factors were also discussed. Limited continuity of care due to staffing shortages and closures of maternity units were mentioned as risk factors:

*‘Yes, it was an absolute no-go for university hospitals, for example, to turn patients away. But we must do it now because we simply can’t guarantee patient safety due to the staffing situation.’* (Expert 2)

Psychological and social influencing factors form a further category. One expert referred to social perceptions of birth:

*‘… and if you are a good mother, then you can manage it without painkillers, without a caesarean section, without an episiotomy. And if things turn out differently, then it’s basically the women’s fault. ... I think it’s also a cultural view, how we look at the woman giving birth. You just have to put up with it, you must get through it. And childbirth is no walk in the park. But afterwards you’ve forgotten everything. It’s only now that you realize that it’s not like that.’* (Expert 1)

The behavior of the caregivers was also named by the experts as a possible factor in the development of traumatic birth experiences. These include communication, boundary violations, and the experience of loss of control. In addition, the importance of a trauma-sensitive birth planning discussion was emphasized, in which a comprehensive anamnesis can take place.

*Measures to improve trauma-sensitive obstetric care*

The measures to promote trauma-sensitive obstetric care can be divided into three central areas:

Structural measures

The experts unanimously emphasized the need for further training to raise awareness and to provide providers with the appropriate skills. It is also essential to create spaces for open exchange. Supervision, debriefings, and case conferences were mentioned as useful measures. The importance of management support for these measures was stressed:

*‘… and it is certainly also important that the management level not only stands behind it rhetorically, but also in terms of content, promotes offers, and creates space for discussions on the topic.’* (Expert 4)

Conceptual measures

One expert presented the ‘5 Es’ concept (self-determination of the birthing person versus the integrity of the child, evidence-based practice, an ethical attitude, and joint decision-making) as a framework for prevention.

Individual measures

All experts emphasized the importance of self-reflection on the part of caregivers. A conscious examination of one’s own actions and critical reflection on one’s own role are essential to avoid traumatic experiences:

*‘… that even if the staff, the midwife, the physicians have good will, good will alone is not enough.’* (Expert 1)

It was also emphasized that every person giving birth could potentially be affected by traumatic experiences. A trauma-sensitive attitude, therefore, means always considering the possibility of previous trauma and communicating in a mindful and respectful manner:

*‘Every woman is potentially affected, not only if I have recognized that this woman is affected. She told me openly, which is rather unlikely, but for every woman to communicate and ask for consent in such a way that you act in a trauma-sensitive manner ...’* (Expert 4)

*Barriers to trauma-sensitive obstetric care*

Both individual and structural reasons were cited as obstacles to the implementation of trauma-sensitive obstetric care. On an individual level, the process of self-reflection and recognition was described as particularly challenging:

*‘It is incredibly painful to realize that you have always tried hard, but that it is not enough. It’s a painful process. … It’s no longer enough to just say “here we go” and that’s an exhausting process. It’s also a painful process in parts.’* (Expert 1)

In addition to these individual challenges, there are also systemic obstacles within obstetrics. Current quality criteria for successful births were criticized for focusing exclusively on physical outcomes, neglecting psychosocial aspects. At a structural level, staffing shortages, discontinuity of care, and lack of remuneration for ‘talking medicine’ were reported as hindering factors.

*Suggested solutions*

To overcome these barriers, experts highlighted two central approaches. First, the systematic integration of trauma-sensitive content into professional training in both medicine and midwifery. Second, the need for ongoing self-reflection and a conscious, trauma-sensitive attitude among providers:

*‘It is unacceptable that this is not a topic in training. So if obstetrics is taught, then it must be addressed. This is a fundamental issue.’* (Expert 1)

## DISCUSSION

This mixed-methods study explored obstetric care providers’ perceptions of traumatic birth experiences and options for optimizing trauma-sensitive care. Across both the survey and interviews, providers emphasized loss of control and the birthing person’s subjective experience as defining features of traumatic birth experiences. While the survey highlighted poor communication and lack of emotional support as key causes, the interviews added structural and societal dimensions, such as staffing shortages, discontinuity of care, and cultural expectations. Regarding preventive measures, all proposed strategies were considered important, with emotional support rated highest. While further training was rated as important in the quantitative survey, its implementation was rated as insufficient. In addition to training, the experts particularly emphasized the self-reflection of caregivers, as training alone was regarded as insufficient, without a trauma-sensitive mindset. Obstacles to trauma-sensitive care were identified on two levels: the survey pointed to structural barriers such as lack of time and staff, while the interviews highlighted individual challenges, particularly the limited capacity for self-reflection among providers. Experts further criticized that current quality criteria in obstetrics focus almost exclusively on physical outcomes, thereby neglecting psychosocial dimensions of birth. The quantitative and qualitative findings converge, highlighting the need for both individual and systemic changes to strengthen trauma-sensitive obstetric care.

Although no statistically significant differences were found between physicians and midwives, some tendencies could be observed. Participation in the study was higher among midwives (n=66) than physicians (n=36), which may indicate a greater sensitivity to the topic within this professional group. Professional experience also appeared to play a role: participants with more years of experience tended to define traumatic births through medical complications, while those with fewer years of experience more often highlighted psychological aspects and expressed stronger agreement with the necessity of trauma-sensitive care. This aligns with the qualitative findings, where experts described trauma-sensitive obstetrics as a relatively new topic that has only recently gained attention in the field. Overall, however, these results should be interpreted as tendencies rather than clear group differences.

The study confirms many findings on the development of traumatic birth experiences and expands them with the specific perspectives of caregivers. That the subjective experience of persons giving birth is a key indicator is consistent with the research^2,10,11^. Several studies have identified the experience of medical complications as a contributing factor to a traumatic birth experience^1,3,6,9,21,30,31^. Although the results of this study also mention medical complications as potentially traumatizing, they also point out that a birth that is objectively perceived as physiological can be subjectively experienced just as traumatically as a highly complicated birth. The question of a new definition of traumatic birth experience has also already been addressed in the literature^31^. In the study, it became clear that the behavior of caregivers can have a significant influence on the development of traumatic birth experiences. This is consistent with existing studies that have analyzed the perspective of those affected. In particular, a lack of communication and a lack of emotional support were named by the caregivers in this study as a central risk factor for a traumatic birth experience, aspects that also play a central role in the perspective of those affected^13,29,30,32^. Continuity of care models, particularly midwifery continuity of care, have been shown to improve women’s emotional wellbeing and birth experience^13,29,33,34^. This study focused on the providers’ perspective in inpatient care, which in Germany typically involves discontinuity of care during birth due to hospital shift systems. Therefore, continuity of care was not directly assessed in the survey. However, its absence was indirectly reflected in participants’ concerns regarding staffing shortages and discontinuity of care, as reported both by survey respondents and by experts in the interviews. The results of this study represent the perceptions of providers rather than the objective causes of traumatic births. Providers perceptions are crucial to a trauma-sensitive care, since providers’ views directly influence how care is delivered, which practices are prioritized, and how trauma-sensitive care principles are integrated into daily routines. By adding providers’ perspectives to the existing research from the viewpoint of birthing persons, it is possible to combine both perspectives to achieve a comprehensive understanding of traumatic birth experiences.

Based on these findings, targeted measures can be derived to make obstetrics more trauma-sensitive:

1. Practice: Trauma-sensitive care requires both skills and attitudes. Training in trauma-sensitive communication and informed consent should be mandatory and supported by regular opportunities for reflection, such as supervision and case conferences.
2. Adaptation of the quality criteria: Validated instruments such as the Childbirth Experience Questionnaire (CEQ-2) or screening tools for postpartum PTSD should be integrated into clinical practice^24,25,35^. These instruments can systematically capture psychological consequences, which providers in this study perceived to be frequently underestimated.
3. Structural improvements: Structural conditions must be addressed to strengthen the framework of the healthcare system, including measures to enhance staffing, reduce time constraints, and promote models of continuity of care.

These options for action aim to steer obstetric care in a direction that not only considers the medical aspects but also focuses on the emotional and psychological needs of the person giving birth. This can create a safe space that prevents traumatic experiences and promotes the well-being of persons giving birth.

### Strengths and limitations

One of the strengths of this study lies in the combination of qualitative and quantitative methods. In addition, the study stands out because it explicitly considers the perspective of professionals and derives options for action, thus complementing the perspective of those affected. However, several limitations should be considered when interpreting the results. Firstly, the sample size was relatively small, and participation was voluntary, which may have introduced selection bias and limited generalizability. Secondly, the qualitative component consisted of four expert interviews and therefore does not aim to reach theoretical saturation but rather provides complementary perspectives to the survey results. Thirdly, the study relied on self-reported perceptions of providers rather than objective measures of traumatic birth experiences. Despite these limitations, the study contributes valuable insights into providers’ perceptions of traumatic birth experiences and highlights potential strategies for improving trauma-sensitive obstetric care.

## CONCLUSIONS

Overall, this study contributes to the topic of trauma-sensitive obstetrics by considering the perspectives of both midwives and physicians and developing concrete recommendations for practice. One of the most important aspects highlighted by the interview participants was the role of self-reflection among healthcare providers. While training in trauma-sensitive communication was considered essential, experts emphasized that knowledge alone is insufficient without a reflective and respectful professional attitude.

Although continuity of care was not directly assessed in this survey, the barriers reported by participants – such as staff shortages and fragmented care structures – suggest that strengthening relational models may represent an important avenue for improving trauma-sensitive obstetric care.

The results show that a comprehensive optimization of obstetric care – at both individual and structural levels – is necessary to prevent traumatic birth experiences.

## Supplementary Material



## Data Availability

The data supporting this research are available from the authors on reasonable request.
