# Supplementary material for: Trauma-sensitive obstetrics: Potential for optimization from the providers’ perspectives
Source: Eur J Midwifery. 2026 Jun 23;10:10.18332/ejm/219322. doi: 10.18332/ejm/219322 (PMC13289673; doi:10.18332/ejm/219322)
Supplement: Supplementary file 1 [file EJM-10-21-s1.pdf]

CHERRIES Checklist

Checklist for Reporting Results of Internet E-Surveys (Eysenbach, 2004)

Manuscript Title: **TRAUMA-SENSITIVE OBSTETRICS - POTENTIAL FOR OPTIMIZATION FROM THE PROVIDERS' PERSPECTIVES**

Author: Louisa Horvath & Silke Pawils

1. Survey design
Described in the section 'Quantitative data collection: online survey'.

2. IRB approval and informed consent process
Described in a special section of the journal editoring system: The study was approved by the ethics committee (LPEK-0804). Informed consent was obtained.

3. Development and pretesting
A pretest was conducted with two care providers (doctor and midwife).

4. Recruitment process and participants
The sample was recruited via study information containing QR codes, distributed in person and by post to 210 maternity clinics across Germany.

5. Survey administration
The survey was technically open-access (no password/token required) via SoSciSurvey, but the distribution was restricted through targeted QR codes handed out in selected clinics.

6. Contact mode
Recruitment information was distributed in person and by post.

7. Advertising the survey
Study information was shared directly with the QR codes in maternity wards.

8. Web/email
The link to the survey was made accessible through QR codes.

9. Mandatory/voluntary

Voluntary survey.

10. Incentives
Participants were offered access to the aggregated survey results.

11. Time/Date
The survey ran from 28.10.2024 to 01.02.2025.

12. Randomization of items or questionnaires
Not applicable – all participants received the same questionnaire.

13. Adaptive questioning
Not used.

14. Number of items
The questionnaire included a total of 58 items.

15. Number of screens/pages
Four pages.

16. Completeness check
Participants were only included if less than 30% of the questionnaire was missing.

17. Review step
No option to review or change answers.

18. Unique site visitor
Not applicable – The survey was distributed via targeted invitations (QR codes/postal distribution), and no view or participation rates were recorded. Duplicate entries were not expected or tracked.

19. View rate
The survey page was accessed 275 times. Because the exact number of invited individuals is unknown, a formal view rate could not be calculated.

20. Participation rate
A total of 275 survey page views were recorded. Of these, 147 participants began the questionnaire, resulting in a participation rate of 53.5%.

21. Completion rate
72% – 102 of 147 participants met inclusion criteria based on response completeness (<30% missing).

22. Handling if incomplete questionnaires
Participants were only included if less than 30% of the questionnaire was missing.

23. Statistical correction
Regression analysis and descriptive statistics were used, including covariates like professional experience.

© 2026 Horvath L. and Pawils S.
